# Nonsurgical Approach to Treat Post‐Rhinoplasty Vascular Complication With Hyaluronic Acid Injection

**DOI:** 10.1111/jocd.70294

**Published:** 2025-06-30

**Authors:** Shuai Qiang, Xing Fan, Yue Yin, Ping Xue, Wen‐Jie Dou, Tong Li, Qing Yang

**Affiliations:** ^1^ Department of Plastic and Reconstructive Surgery Xijing Hospital, Forth Military Medical University Xi'an Shaanxi China

**Keywords:** early intervention, hyaluronic acid fillers, hyaluronidase therapy, skin necrosis, vascular compromise

## Abstract

**Background:**

Hyaluronic acid (HA) fillers are popular for their minimally invasive nature and immediate aesthetic outcomes, but vascular compromise remains a significant complication. This study investigates optimized therapeutic strategies to prevent tissue necrosis following HA filler injections.

**Aims:**

To evaluate the efficacy of two treatment regimens for managing impending nasal skin necrosis due to HA filler injections, focusing on preventing tissue necrosis and reducing complications such as pigmentation changes, scarring, and telangiectasia.

**Patients and Methods:**

A retrospective analysis was conducted on 41 female patients (mean age: 36.2 years) treated at a single referral center between 2018 and 2023. Patients presented within 72 h after developing ischemic symptoms following HA injections for nasal augmentation. Two treatment regimens, both integrating hyperbaric oxygen therapy (HBOT), epidermal growth factor (EGF) gel, and corticosteroids, were evaluated: one using nitroglycerin (NTG) and the other using isosorbide dinitrate (ISDN).

**Results:**

All patients achieved complete wound healing, with no significant differences in scar formation or hypotension between the treatment groups (*p* > 0.05). Hyaluronidase (HSE) was administered externally at referring clinics prior to hospital admission in 31.7% of patients (without subsequent standardized HSE protocol), significantly reducing telangiectasia incidence (*p* < 0.05) but not affecting scarring or pigmentation (*p* > 0.05). Early hospital presentation (< 48 h) was associated with lower pigmentation changes (*p* < 0.05) but did not significantly affect scarring. Residual complications included scar formation in 9 of 41 patients (21.9%, 95% CI 12.0–36.7), telangiectasia in 16 of 41 (39.0%, 95% CI 25.7–54.3), and pigmentation changes in 22 of 41 (53.7%, 95% CI 38.7–67.9).

**Conclusions:**

Combined therapies effectively manage HA‐induced vascular compromise, with early intervention critical for reducing ischemic complications and improving patient outcomes.

## 1 Introduction

The demand for soft tissue fillers as a minimally invasive approach to facial rejuvenation has significantly increased over the past decade. By 2023, the global market for dermal fillers reached approximately USD 5.08 billion, with projections indicating growth up to USD 10.16 billion by 203 [1]. The appeal of these fillers is primarily driven by their ease of application, immediate visible improvements, and relatively low complication rates. Among these fillers, nonsurgical rhinoplasty (NSR) using hyaluronic acid (HA) has gained considerable popularity due to its minimally invasive nature, predictability, and minimal recovery time [2, 3]. Although reported vascular complications associated with HA fillers are low (0.001%–0.005%) [4], widespread use has inevitably led to an increase in severe complications, mainly due to inadvertent intravascular injections or external venous compression [5].

Recent studies on embolic pathways reveal that nasal filler emboli predominantly propagate through the anastomoses between the angular artery and anterior ethmoidal artery, posing risks of retrograde occlusion in ocular and cerebral circulations [6]. Practitioners are therefore encouraged to possess comprehensive knowledge of facial vascular anatomy, maintain vigilance for early ischemic signs, and initiate prompt intervention to prevent severe outcomes such as skin necrosis, vision impairment, or cerebral infarction [7]. According to the Manufacturer and User Facility Device Experience (MAUDE) database, skin necrosis accounts for 3.5% of adverse events related to fillers, with frequent involvement of the nasolabial folds (20.8%) and nose (15.6%) [8].

Clinically, vascular embolism from HA fillers is characterized by significant pain and distinctive ischemic discoloration at the injection site, often delayed by the presence of lidocaine [9]. If not rapidly diagnosed and treated, ischemic injuries can escalate to severe necrosis, ulceration, and irreversible tissue damage, resembling damage seen in compromised skin flaps [10]. In countries like China, the proliferation of practitioners lacking proper medical training and anatomical understanding has contributed to an increase in complications, often resulting in prolonged recovery and substantial cosmetic sequelae, including prominent pigmentation and hypertrophic scarring.

Hyaluronidase (HSE) remains the established gold‐standard treatment for ischemic complications associated with HA fillers due to its rapid dissolution of filler material and restoration of tissue perfusion when administered promptly. Although occasional concerns have been raised about its use in retrobulbar vascular incidents, HSE continues to be the cornerstone therapy for acute ischemic complications in soft tissues.

This study aims to identify optimal treatment strategies for managing impending skin necrosis following nasal augmentation procedures using HA fillers. By retrospectively analyzing the clinical outcomes of 41 consecutive patients, we aim to provide valuable insights into the diagnosis, management, and prevention of tissue necrosis associated with HA filler injections.

## 2 Patients and Methods

Between May 2018 and July 2023, 41 female patients (mean age 36.2 years, range 25–46 years) who presented within 72 h following nasal ischemia from HA filler injections were enrolled in this retrospective study (Table 1). Patient demographics, clinical history, injection sites, symptoms, treatments, and outcomes were thoroughly reviewed using previously collected, fully de‐identified medical records, involving no additional risk or interventions. Consequently, this study was exempt from formal institutional review board oversight and complied with the Declaration of Helsinki ethical standards. All patients received HA injections externally at various clinics, predominantly involving the nasal dorsum, nasal tip, nasal ala, and columella. HA dosage details were unavailable.

**TABLE 1:** Demographics and clinical features (injection sites, needle types, and symptoms).

	*N* (%)
Total number	41 (full skin necrosis)
Gender	
Female	41 (100%)
Age	
Mean	36.2
Range	25–46
Injection site (115 injection sites for full skin necrosis)	
Nasal dorsum	41 (100%)
Nasal tip	36 (87.8%)
Nasal ala	6 (14.6%)
Columella	8 (19.5)
Needle type	
Needle	33 (80.5%)
Cannula	8 (19.5%)
Symptoms (symptoms immediately or subsequently occurred after filler injection)	
Significant pain	41 (100%)
Skin change (“spotted” or “map‐like” changes)	41 (100%)
Bleeding	4 (9.8%)
Pallor and Swelling	41 (100%)
Numbness	22 (53.7%)
Time from injection to hospital admission (h)	
< 12 h	4 (9.8%)
12–24 h	4 (9.8%)
25–36 h	1 (2.4%)
37–48 h	3 (7.3%)
49–60 h	3 (7.3%)
61–72 h	26 (63.4%)
Hyaluronidase treatment prior to hospital admission	
Yes	13 (31.7%)
No	28 (68.3%)
Clinical outcomes of the complications	
Scar	9
Pigmentation	22
Complete recovery	41 (100%)
Improved	0
Noneffective	0
Tissue defect	0
Telangiectasia	16 (39.0%)

Clinical symptoms included pallor, swelling, significant pain, numbness, bleeding at the injection site, and characteristic “map‐like” skin lesions. No ocular symptoms were reported. Patients were categorized into two therapeutic groups: Group A (*n* = 20) received nitroglycerin (NTG), hyperbaric oxygen therapy (HBOT), epidermal growth factor (EGF) gel, and dexamethasone; Group B (*n* = 21) received isosorbide dinitrate (ISDN) with identical supportive therapies. HBOT protocol involved daily administration of 100% oxygen at 2.0 ATA, including a 15‐min compression phase, 60 min of stable‐pressure oxygen inhalation with a 10‐min air interval halfway, and gradual decompression over 15 min. Treatment duration was 1–2 weeks, depending on severity.

**FIGURE 1:**
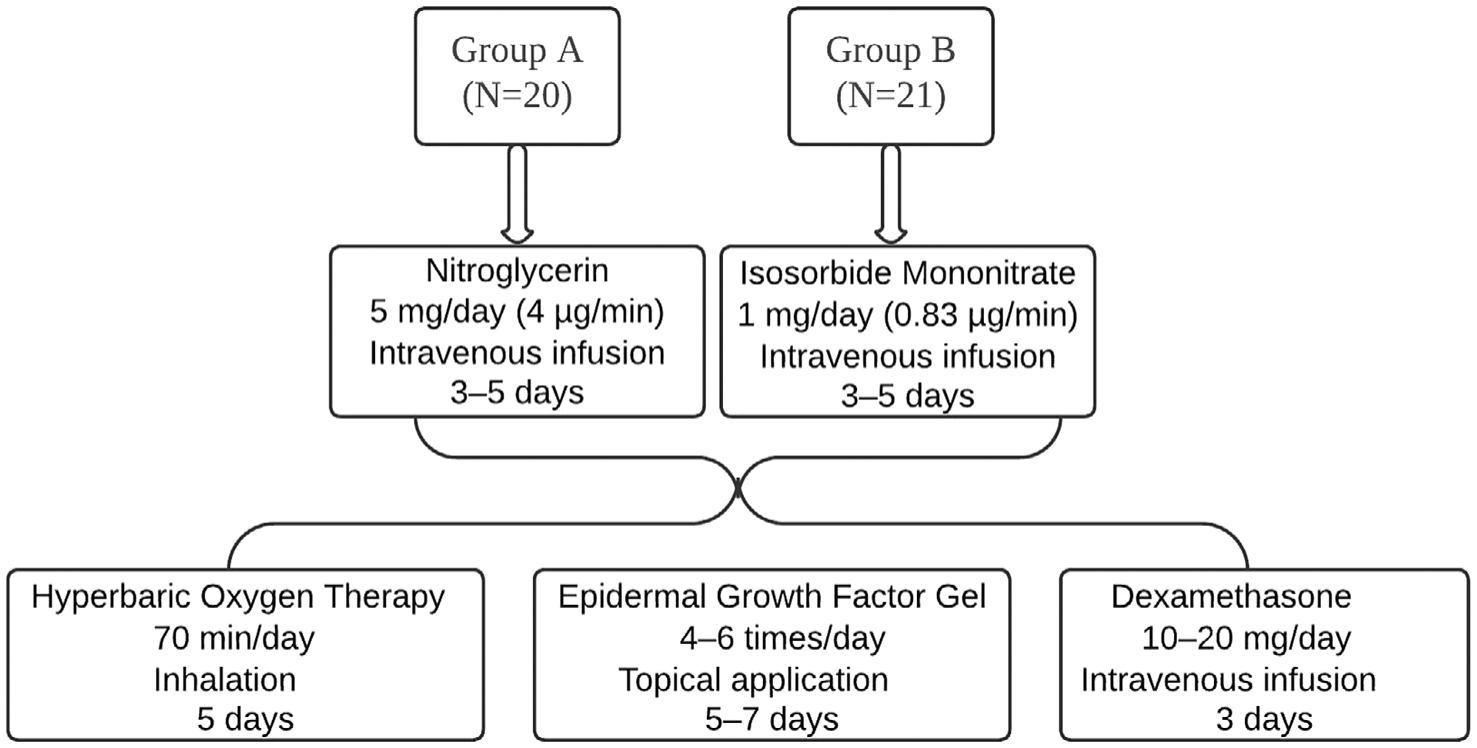
Treatment protocols for managing nasal vascular compromise following hyaluronic acid filler injection. Patients were divided into two treatment groups: Group A (*n* = 20) received nitroglycerin (5 mg/day via intravenous infusion for 3–5 days), while Group B (*n* = 21) received isosorbide mononitrate (1 mg/day via intravenous infusion for 3–5 days). Both groups were treated with adjunctive therapies, including hyperbaric oxygen therapy (70 min/day for 5 days), epidermal growth factor (EGF) gel (topical application 4–6 times/day for 5–7 days), and dexamethasone (10–20 mg/day via intravenous infusion for 3 days).

**FIGURE 2:**
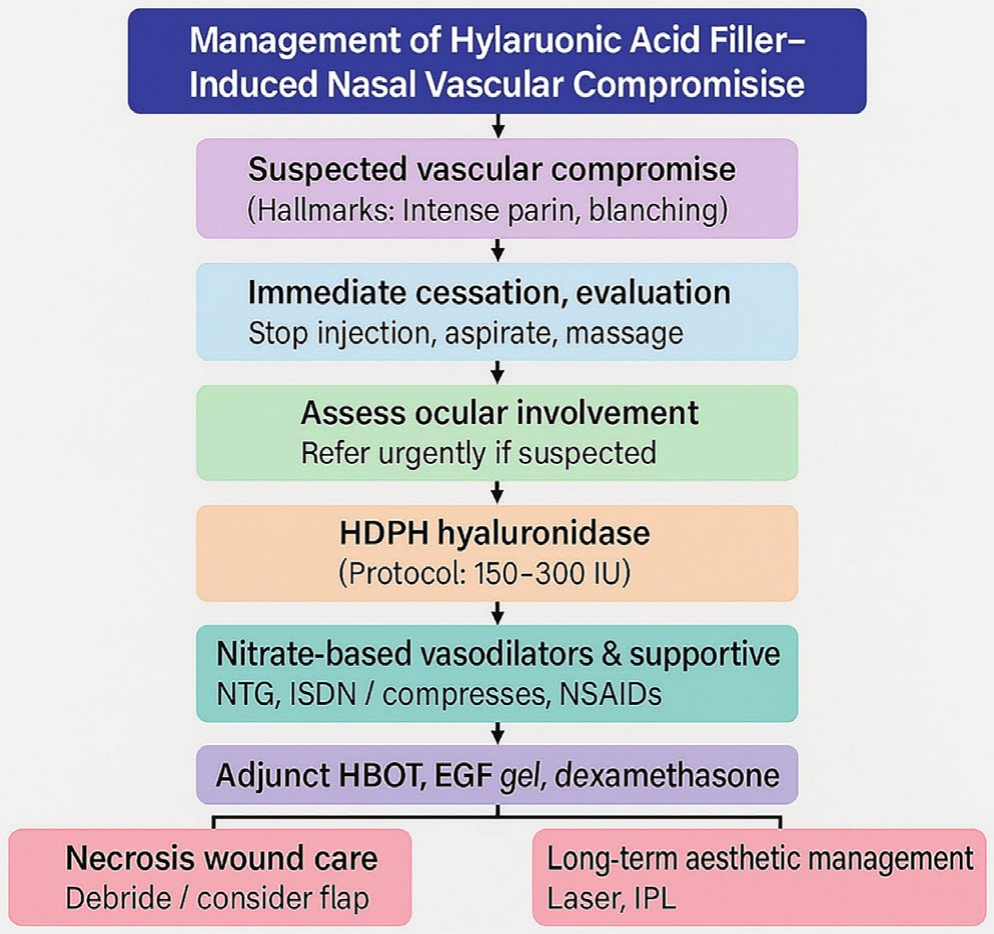
Algorithm for management of hyaluronic‐acid filler–induced nasal vascular compromise. The flowchart outlines (i) early recognition, (ii) immediate cessation and evaluation, (iii) ocular assessment, (iv) high‐dose pulsed hyaluronidase (HDPH) injection, (v) nitrate‐based vasodilator and supportive therapy, (vi) adjunct hyperbaric oxygen, epidermal growth factor gel, and corticosteroids, (vii) monitoring for reperfusion, and (viii) pathways for necrosis debridement and long‐term aesthetic management.

Patients provided informed consent and were advised of potential complications such as refractory wounds, long‐term treatment, nasal deformity, and hypertrophic scarring. Treatment protocols combining pharmacological and supportive therapies were individualized to optimize outcomes. Therapeutic interventions and a management algorithm for HA filler‐induced vascular compromise are detailed in Figures 1 and 2, respectively.

Outcomes assessed 1 week post‐treatment were classified as complete epithelial closure (fully re‐epithelialized, intact skin), improved (reduced size without complete healing), or ineffective (persistent or expanding wounds). Adverse events and skin recovery outcomes, including scarring, pigmentation changes, and telangiectasia, were documented meticulously. Statistical analyses utilized SPSS 21.0 software (IBM Corp., Armonk, NY, USA), employing chi‐square tests for categorical variables. A *p* value < 0.05 indicated statistical significance.

## 3 Results

Detailed patient demographics and clinical characteristics are summarized in Table 1. All enrolled patients were female (*n* = 41), with a mean age of 36.2 years (range: 25–46 years). A total of 115 distinct injection sites were analyzed; the nasal dorsum was universally involved (41/41, 100%), followed by the nasal tip (36/41, 87.8%), columella (8/41, 19.5%), and nasal ala (6/41, 14.6%). Sharp needles were predominantly used for filler injection (33/41, 80.5%), whereas blunt cannulas were employed in fewer instances (8/41, 19.5%).

Clinical symptoms consistently reported across all patients included significant pain, pallor, swelling, and characteristic “spotted” or “map‐like” ischemic skin discolorations (41/41, 100%). Numbness was experienced by over half of the cohort (22/41, 53.7%), and bleeding at the injection site was comparatively uncommon, affecting only 9.8% (4/41). Time intervals from initial filler injection to hospital presentation varied notably, with the majority of patients presenting between 61 and 72 h (26/41, 63.4%), and smaller proportions within 12 h (4/41, 9.8%), between 12–24 h (4/41, 9.8%), 37–48 h (3/41, 7.3%), 49–60 h (3/41, 7.3%), and 25–36 h (1/41, 2.4%).

Externally administered HSE, without subsequent standardized dosing protocols, was provided prior to hospital admission in 31.7% (13/41) of patients. During hospitalization, transient hypotension occurred in three patients (7.3%), managed conservatively without altering overall treatment timelines or outcomes.

**FIGURE 3:**
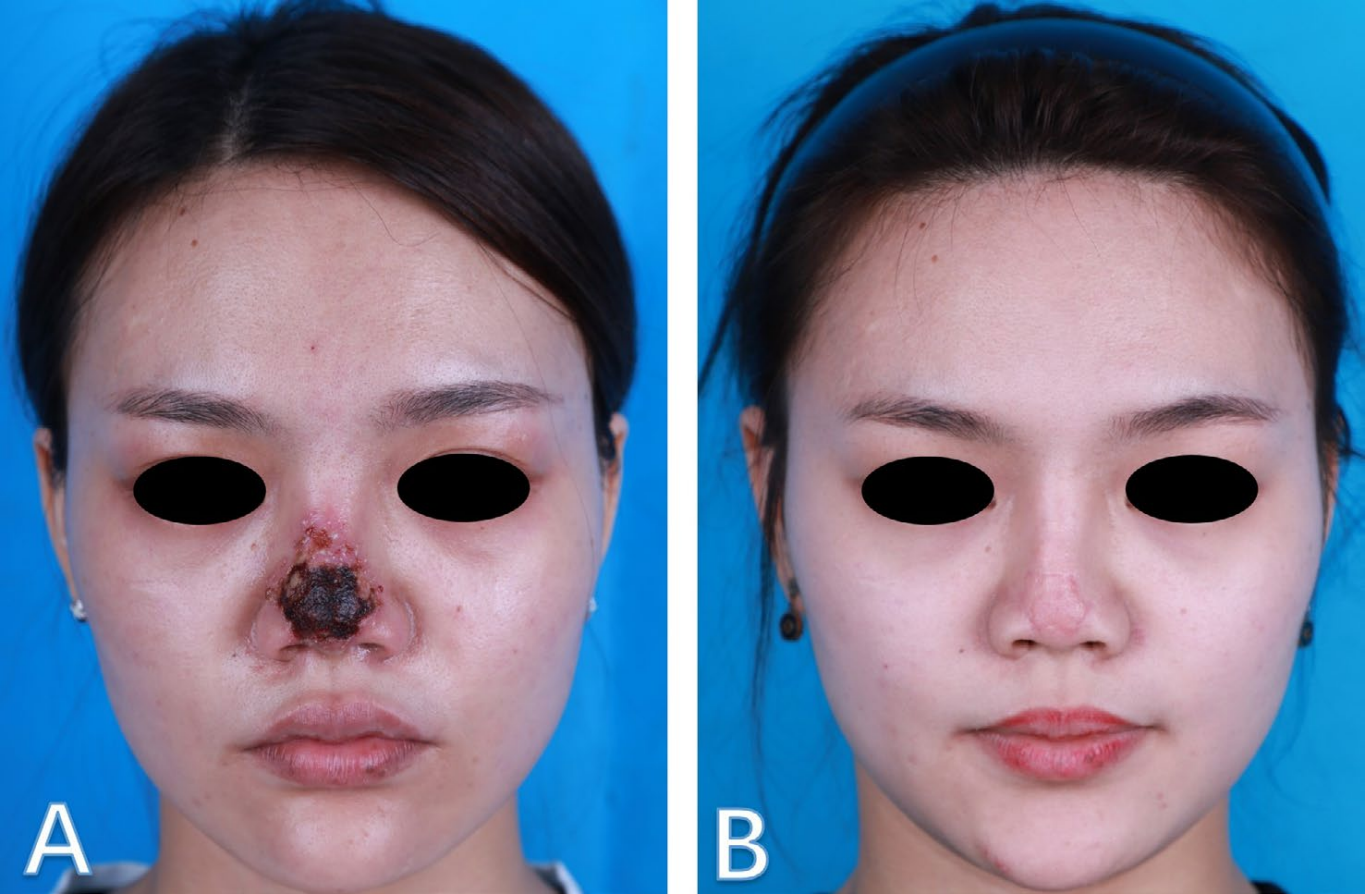
Patient 1: (A) Necrosis of the nasal tip, covered by eschar, occurring 3 days post‐injection. (B) The patient was treated with isosorbide dinitrate (ISDN), HBOT, EGF gel, and dexamethasone without the use of hyaluronidase, following 9 months of conservative wound care, showing improvement after the treatment regimen.

**FIGURE 4:**
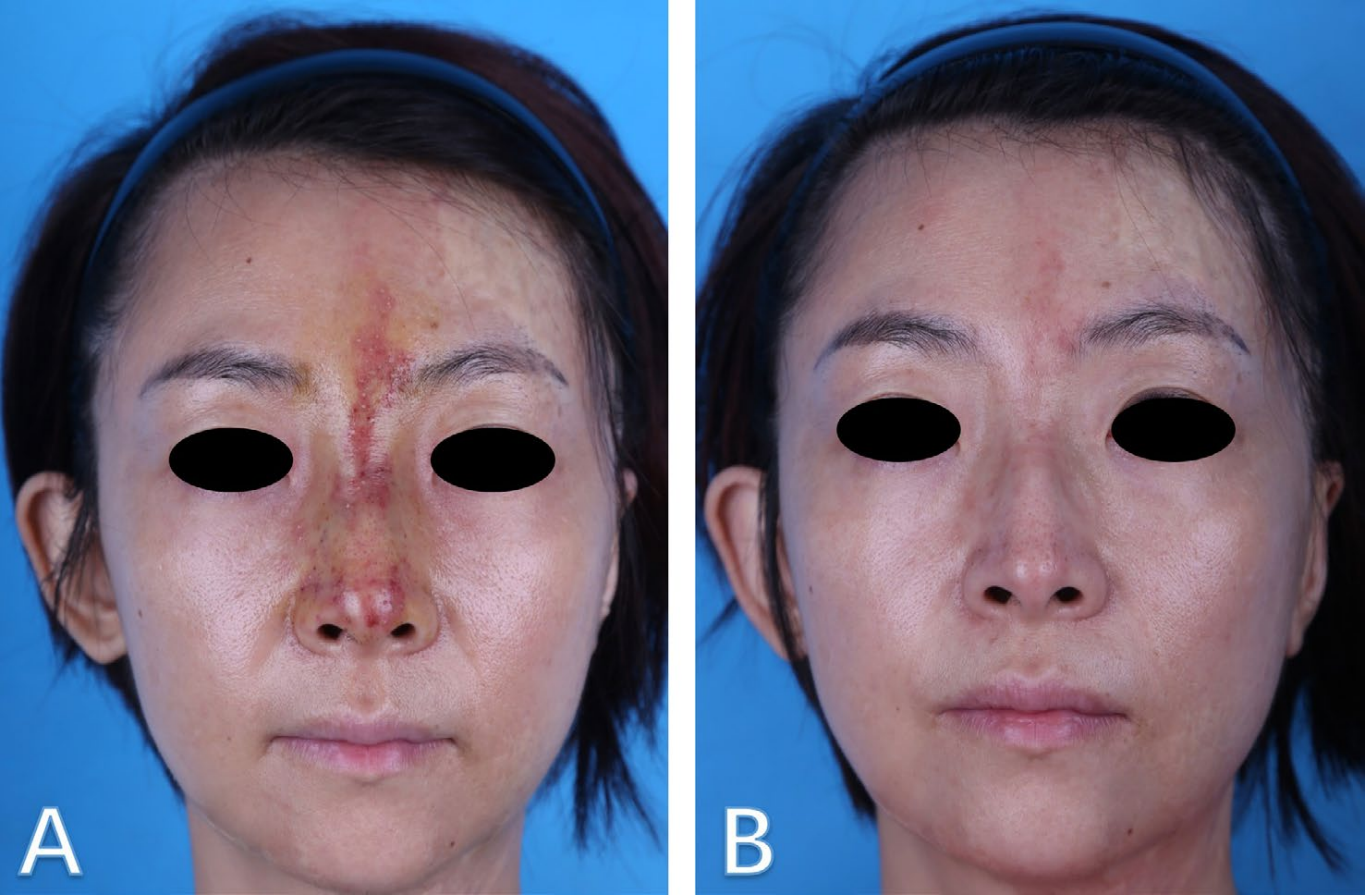
Patient 2: (A) Photographic documentation taken 70 h post‐injection of hyaluronic acid, showing a vascular complication involving the nasal region with evidence of intravascular involvement. (B) The patient was treated with nitroglycerin (NTG), hyperbaric oxygen therapy (HBOT), epidermal growth factor (EGF) gel, and dexamethasone, with subsequent improvement observed 10 days after initiation of treatment.

All patients (41/41, 100%) achieved complete epithelial wound closure (Figures 3 and 4). Despite full healing, residual aesthetic complications were frequent: post‐inflammatory pigmentation developed in 53.7% (22/41), telangiectasia in 39.0% (16/41), and visible scarring in 21.9% (9/41). No patient experienced persistent open wounds or irreversible tissue defects.

Comparative outcomes between the two nitrate‐based treatment protocols (Table 2) revealed no significant differences regarding scar formation or hypotension events. Specifically, scar formation was noted in four patients treated with nitroglycerin (NTG; Group A) and five patients treated with isosorbide dinitrate (ISDN; Group B). Hypotension occurred in two patients in Group A and one patient in Group B, with statistical analysis (Chi‐square test) confirming no significant difference in efficacy or safety profiles between groups (*p* > 0.05).

**TABLE 2:** Comparison of prognostic outcomes and adverse events between two nitrate‐based treatment groups for nasal vascular compromise.

Group	Number of cases (*n*)	Dosage (mg) and duration (days)	Scar (*n*)		Hypotension (*n*)	
			Present	Absent	Present	Absent
Nitroglycerin—Group A	18	5 mg/day × 3 days, 4 μg/min	4	14	2	16
Isosorbide Dinitrate—Group B	23	1 mg/day × 3 days, 0.83 μg/min	5	18	1	22
*p*			> 0.05		> 0.05	

Overall residual complication rates (Table 3) were 21.9% (95% CI, 12.0–36.7) for scarring, 39.0% (95% CI, 25.7–54.3) for telangiectasia, and 53.7% (95% CI, 38.7–67.9) for pigmentation changes.

**TABLE 3:** Relationship between hyaluronidase use, hospital presentation time, and prognosis in patients with nasal vascular compromise.

Time to hospital presentation	Total patients (*n*)	Hyaluronidase injection cases (*n*)		Scar (*n*)		Pigmentation (*n*)		Telangiectasia (*n*)	
		Injected	Not injected	With	Without	With	Without	With	Without
< 12 h	4	1	3	0	4	0	4	3	1
12–24 h	4	4	0	2	2	0	4	0	4
25–36 h	1	1	0	0	1	0	1	0	1
37–48 h	3	2	1	0	3	3	0	1	2
49–60 h	3	0	3	0	3	0	3	3	0
61–72 h	26	5	21	7	19	19	7	9	17
Total	41	13	28	9	32	22	19	16	25

Statistical analyses further revealed that external administration of HSE significantly reduced the incidence of telangiectasia (*p* < 0.05), likely reflecting improved microvascular recovery and reduced endothelial damage (Table 4). However, there was no statistically significant impact of HSE administration on scar formation (*p* > 0.05).

**TABLE 4:** Relationship between hyaluronidase use combined with comprehensive therapy and prognosis.

Hyaluronidase injection	Scar (*n*)		Telangiectasia (*n*)	
	With	Without	With	Without
With	6	22	15	13
Without	3	10	1	12
*p*	> 0.05		< 0.05	

A notable association was identified between early hospital presentation (within 48 h of filler injection) and reduced incidence of pigmentation changes. Patients admitted early demonstrated significantly fewer pigmentation complications (3/12, 25.0%) compared with those admitted after 48 h (19/29, 65.5%; *p* < 0.05). Although scar formation was less common among patients admitted earlier (2/12, 16.7%) compared with later admissions (7/29, 24.1%), this difference did not reach statistical significance (*p* > 0.05). Similarly, no significant association was found between timing of hospital presentation and incidence of telangiectasia (*p* > 0.05; Table 5).

**TABLE 5:** Relationship between time to hospital presentation and prognosis.

Time from injection to hospital admission (h)	Cases (*n*)	Scar (*n*)		Pigmentation (*n*)		Telangiectasia (*n*)	
		With	Without	With	Without	With	Without
≤ 48 h	12	2	10	3	9	4	8
49–72 h	29	7	22	19	10	12	17
Total patients (*n*)	41	9	32	22	19	16	25
*p*		> 0.05		< 0.05		> 0.05	

Collectively, these results underscore the protective effects of prompt intervention, particularly with regard to reducing pigmentation complications, and suggest potential vascular benefits associated with early HSE application. However, the relationship between early intervention and scar formation remains inconclusive, necessitating additional research into optimized therapeutic windows and adjunctive treatment protocols for long‐term aesthetic outcomes.

## 4 Discussion

The nasal structure significantly contributes to facial aesthetics, serving as the central element in achieving harmonious facial proportions. Modification of nasal features—such as addressing a flat or poorly defined dorsum, a low radix, an underprojected nasal tip, or recessed columella—can profoundly improve facial contour, especially in Asian populations characterized by subtler nasal profiles compared to Caucasians [11]. While surgical rhinoplasty remains the gold standard for comprehensive nasal correction, NSR with HA fillers has rapidly become popular due to its minimally invasive nature, predictable outcomes, affordability, and minimal downtime [12, 13, 14].

Although HA‐based NSR is generally safe and efficacious, increasing procedural volume and variability in practitioner expertise have contributed to the rise of severe complications, notably vascular compromise [15, 16, 17].

Although HA‐based NSR is generally safe and efficacious, increasing procedural volume and variability in practitioner expertise have contributed to the rise of severe complications, notably vascular compromise [15, 16, 17]. The relatively low incidence of vascular occlusion (0.001%–0.005%) [4] belies the gravity of potential outcomes, including ischemic necrosis and, rarely, vision impairment or cerebral infarction [6]. These complications typically arise from inadvertent intra‐arterial injections or extrinsic venous compression and manifest clinically with significant pain, pallor, swelling, and characteristic map‐like discoloration [9, 18]. Consistent with previous reports [2, 15], these hallmark symptoms were uniformly observed among patients in our study.

The pathophysiology of severe vascular compromise predominantly involves embolic propagation via rich anastomoses between the angular artery and anterior ethmoidal artery, posing risks of retrograde ocular and cerebral ischemia [6]. Thus, comprehensive anatomical knowledge, proper injection technique, and vigilant patient monitoring are paramount. Although blunt cannulas, aspiration techniques, and slow, controlled injections are recommended safety strategies, none completely eliminate risk [2]. In our study, despite a minority (19.5%) receiving cannula‐based injections, severe ischemic complications persisted, underscoring the multifactorial nature of vascular injury in filler procedures.

A key determinant in the occurrence of these complications is the skill and experience of the clinician. Proper knowledge of facial anatomy, appropriate filler selection, and mastery of injection techniques are essential to minimize the risks of vascular compromise. Strategies such as the use of blunt cannulas, slow and controlled injection techniques, and aspiration prior to injection are commonly recommended to reduce vascular risks. However, it should be noted that the reliability of aspiration remains controversial, as false‐negative aspiration results can occur. Consequently, aspiration should not be relied upon as the sole preventive measure; instead, a combination of multiple safety strategies is advisable [2]. In our study, a small proportion of patients (19.5%) were injected using cannulas, which may reduce the risk of vascular injury compared to traditional needle injections. Despite this, the most severe complication was soft tissue necrosis, often resulting from direct vessel injury or venous compression in the subcutis due to excessive filler placement. HSE, an enzyme that breaks down HA, is commonly used as a treatment for these adverse events [19, 20]. When administered promptly, HSE can dissolve the filler and potentially prevent further tissue damage. However, its effectiveness in reversing advanced ischemic damage is limited, and tissue necrosis may continue in cases where the damage is extensive.

In China, the lack of regulation surrounding aesthetic procedures, especially NSR, has contributed to a rise in the number of poorly trained practitioners performing these injections. This has led to a notable increase in patients seeking treatment for vascular complications resulting from filler injections. Delays in seeking treatment often lead to more complex and difficult‐to‐manage complications. The Complications in Medical Aesthetics Collaborative (CMAC) has reported a surge in cases involving vascular events that require more advanced and extended treatment protocols. These cases, often presenting later in the course of the complication, may not be amenable to simple HSE injection alone [21].

Ischemic tissue caused by a HA filler vascular event can break down quickly, and wound healing could be delayed if vascular perfusion is reversed slowly [9]. Meanwhile, the potential risk of ischemia–reperfusion injury may exist after HSE injection in the treatment of vascular embolism, which could further aggravate tissue damage [22]. Severe ischemic injuries and formation of secondary wounds may require escalation to secondary care for surgical management [9].

Recent studies on vascular compromise following cosmetic injections primarily focus on the mechanisms of vascular embolization and the management of recanalization. However, there is a limited body of research addressing the treatment of secondary wounds resulting from injection‐related complications. While early literature noted the absence of consensus on hyaluronidase dosing for HA filler–induced vascular events, the High‐Dose Pulsed Hyaluronidase (HDPH) protocol introduced by Delorenzi et al. [23] has since gained broad acceptance as a standard treatment regimen. In this study, we present a series of 41 consecutive patients who developed impending nasal skin necrosis after HA nose augmentation. Through retrospective case–control analysis, we review the clinical outcomes and describe our treatment strategies, which aim to contribute to the understanding, diagnosis, and management of such complications, with a focus on preventing tissue necrosis.

HSE is known for its efficacy in dissolving HA fillers and preventing tissue necrosis [16]. However, in cases where tissue damage is extensive, HSE may not prevent further breakdown, as irreversible injury to the underlying structures may already have occurred [9]. While many clinicians recommend HSE injections as an emergency intervention [24], uncertainties persist regarding the optimal injection method, dosage, and efficacy, especially for patients who delay seeking treatment [25]. Previous studies have shown that while HSE can limit the extent of necrosis, it is less effective in resolving large, deep necrotic areas, which often indicate that the wound has entered the inflammatory stage [9]. In such cases, additional therapies aimed at promoting wound healing may be necessary after recanalization.

Treatment modalities such as HBOT and nitrate‐based treatments are frequently used to manage HA occlusion. However, inconsistent application of these treatments can lead to complications, including skin blisters, epidermal shedding, and mild dermal scarring or pigmentary changes. Historically, mild scarring has been regarded as an acceptable outcome. Based on our experience with 41 patients, we propose a comprehensive, systematic treatment plan designed to avoid these complications. This plan includes the use of NTG/ISDN, HBOT, EGF gel, and dexamethasone. For patients with delayed necrosis, this approach effectively improves tissue condition and minimizes scarring. Both nitrate‐based protocols achieved complete epithelial closure in every case, with no inter‐group difference in subsequent scar formation or hypotension. However, pigmentary and vascular sequelae remained prevalent, underscoring the need for long‐term aesthetic management. Based on our experience with 41 patients, both nitrate‐based protocols achieved comparable efficacy and safety, with all patients ultimately attaining complete epithelial closure and no significant differences in scar formation or hypotension between groups. However, the retrospective and combined‐modality nature of this analysis precludes definitive conclusions about the individual contribution of NTG or ISDN to clinical outcomes. Moreover, previous animal investigations have reported potential exacerbation of ischemic injury with topical nitroglycerin, underscoring the need for cautious interpretation [26]. Consequently, while our findings support the inclusion of nitrate therapy within a comprehensive management plan for filler‐related vascular events, larger, prospective studies are essential to isolate the specific effects of nitrates, optimize dosing strategies, and confirm long‐term safety.

Studies have shown that early intervention, particularly within the first 48 h, leads to better outcomes, including complete recovery from complications [2]. When treatment is initiated within 2 days, the ischemic damage is often reversible within a week, with no lasting sequelae. However, delays beyond 48 h increase the likelihood of irreversible ischemia and tissue necrosis, particularly in deeper skin layers. Although HSE can resolve embolic occlusion and alleviate ischemic damage, it cannot repair necrotic tissue.

Endothelial repair following ischemic insult requires activation of angiogenic pathways and extracellular matrix remodeling, processes that HSE does not facilitate. Therefore, in advanced lesions, adjunctive measures—such as growthfactor‐enriched dressings, topical or systemic agents that promote neovascularization, and hyperbaric oxygen therapy—become critical to support microvascular regeneration and wound healing.

Among the patients in this study, 31.7% had previously received HSE injections at other facilities, but the time interval between the injection and vascular occlusion was not consistently documented. Statistical analysis revealed that HSE treatment significantly reduced the incidence of telangiectasia (*p* < 0.05), supporting its role in improving vascular recovery. While our observations suggest that prior external administration of single‐dose HSE was associated with reduced telangiectasia, the absence of a standardized HSE protocol precludes direct comparisons with our nitrate‐based comprehensive management. Therefore, further prospective studies implementing standardized HSE protocols are necessary to definitively determine its efficacy in preventing structural tissue damage and related complications.

Additionally, we observed that early hospital presentation (within 48 h) was associated with a significantly lower incidence of pigmentation changes (*p* < 0.05), highlighting the importance of prompt intervention. Early admission allows for the rapid resolution of ischemic inflammation, thereby reducing the risk of long‐term skin discoloration. Although earlier presentation correlated with a lower incidence of scarring, this difference did not reach statistical significance (*p* > 0.05). These findings underscore the critical role of early detection and treatment in improving patient outcomes. Despite achieving complete epithelial closure in all patients, residual complications, including pigmentation changes (53.7%), scarring (21.9%), and telangiectasia (39.0%), were noted. These sequelae pose significant challenges to patients' aesthetic outcomes and overall quality of life. The higher incidence of pigmentation changes in patients with delayed treatment further emphasizes the need for increased awareness and education regarding the early signs of vascular compromise. For patients with persistent post‐inflammatory hyperpigmentation, chemical peels may be employed, and if unsuccessful, more advanced treatments such as intense pulsed light (IPL), pulsed dye laser (PDL), or fractional lasers may be considered. Permanent telangiectasias can be treated with IPL or PDL therapies.

## 5 Limitations

This retrospective study has several inherent limitations, including its single‐center design and exclusive inclusion of female patients, limiting generalizability. Standardized procedural details such as exact injection depths and anatomical reference points were lacking, precluding detailed analysis of technique‐specific risks. The absence of validated objective assessment scales and standardized photographic documentation further constrained rigorous outcome evaluations. Future prospective studies should include broader, more diverse cohorts with comprehensive standardized data collection to strengthen external validity.

## 6 Conclusion

Timely, protocol‐driven management of filler‐related vascular compromise reliably prevented tissue loss in this cohort, yet substantial aesthetic sequelae persisted. Early intervention curbed necrosis, but post‐inflammatory dyschromia, telangiectasia, and scarring remained common, underscoring the need for long‐term surveillance and adjunctive therapies. Future prospective studies with larger samples should refine therapeutic algorithms and delineate predictors of residual cutaneous morbidity.

## Author Contributions

Shuai Qiang, Xing Fan, Yue Yin, and Qing Yang contributed to the study design, data analysis, and manuscript writing. Ping Xue, Wen‐Jie Dou, and Tong Li conducted patient evaluations, data collection, and treatment administration. Shuai Qiang and Qing Yang provided statistical analysis and interpretation of the results. Shuai Qiang and Qing Yang helped with the study coordination and manuscript revision.

## Ethics Statement

This study was conducted in accordance with the ethical principles outlined in the Declaration of Helsinki. All patients provided written informed consent for their participation in the study, and the research was approved by the Ethics Committee of Xijing Hospital. The study adhered to all privacy and confidentiality guidelines.

## Conflicts of Interest

The authors declare no conflicts of interest.

## Data Availability

The data that support the findings of this study are available on request from the corresponding author. The data are not publicly available due to privacy or ethical restrictions.

## References

[1] Fortune Business Insights , “DFMS, Share & Industry Analysis,” accessed October 28, 2024, https://www.fortunebusinessinsights.com/industry‐reports/dermal‐fillers‐market‐100939.

[2] Z. S. Sun , G. Z. Zhu , H. B. Wang , et al., “Clinical Outcomes of Impending Nasal Skin Necrosis Related to Nose and Nasolabial Fold Augmentation With Hyaluronic Acid Fillers,” Plastic and Reconstructive Surgery 136, no. 4 (2015): 434e–441e. 26397262 10.1097/PRS.0000000000001579

[3] T. C. Kontis , “The Art of Camouflage: When Can a Revision Rhinoplasty be Nonsurgical?,” Facial Plastic Surgery 34, no. 3 (2018): 270–277. 29857337 10.1055/s-0038-1653989

[4] J. L. Cohen , B. S. Biesman , S. H. Dayan , et al., “Treatment of Hyaluronic Acid Filler‐Induced Impending Necrosis With Hyaluronidase: Consensus Recommendations,” Aesthetic Surgery Journal 35, no. 7 (2015): 844–849. 25964629 10.1093/asj/sjv018

[5] V. C. Doyon , C. Liu , R. Fitzgerald , et al., “Update on Blindness From Filler: Review of Prognostic Factors, Management Approaches, and a Century of Published Cases,” Aesthetic Surgery Journal 44, no. 10 (2024): 1091–1104. 38630871 10.1093/asj/sjae091

[6] F. Zhao , Y. Chen , D. He , X. You , and Y. Xu , “Disastrous Cerebral and Ocular Vascular Complications After Cosmetic Facial Filler Injections: A Retrospective Case Series Study,” Scientific Reports 14, no. 1 (2024): 3495. 38347086 10.1038/s41598-024-54202-w PMC10861540

[7] M. King , L. Walker , C. Convery , et al., “Management of a Vascular Occlusion Associated With Cosmetic Injections,” Journal of Clinical and Aesthetic Dermatology 13, no. 1 (2020): e53–e58. PMC7028373 32082474

[8] M. Xiong , C. Chen , Y. Sereda , L. Garibyan , M. Avram , and K. C. Lee , “Retrospective Analysis of the MAUDE Database on Dermal Filler Complications From 2014–2020,” Journal of the American Academy of Dermatology 87, no. 5 (2022): 1158–1160. 35202776 10.1016/j.jaad.2022.02.029

[9] A. Farmer , G. Murray , B. Croasdell , E. Davies , C. Convery , and L. Walker , “Facial Vascular Events and Tissue Ischemia: A Guide to Understanding and Optimizing Wound Care,” Journal of Clinical and Aesthetic Dermatology 14, no. 12 Suppl 1 (2021): S39–S48. PMC8903221 35291261

[10] X. Wang , Q. Zhao , X. Chen , et al., “Use of Liquid Concentrated Growth Factor in the Management of Necrotic Tissue After Facial Vascular Complications Induced by Hyaluronic Acid Injection,” Wounds: A Compendium of Clinical Research and Practice 34, no. 11 (2022): 263–268. 36322917

[11] P. Kim and J. T. Ahn , “Structured Nonsurgical Asian Rhinoplasty,” Aesthetic Plastic Surgery 36, no. 3 (2012): 698–703. 22350307 10.1007/s00266-012-9869-2

[12] T. C. Kontis , “Nonsurgical Rhinoplasty,” JAMA Facial Plastic Surgery 19, no. 5 (2017): 430–431. 28492936 10.1001/jamafacial.2017.0701

[13] A. Manafi , Z. S. Hamedi , A. Manafi , A. Rajabiani , A. Rajaee , and F. Manafi , “Injectable Cartilage Shaving: An Autologous and Long Lasting Filler Material for Correction of Minor Contour Deformities in Rhinoplasty,” World Journal of Plastic Surgery 4, no. 2 (2015): 93–100. 26284177 PMC4537600

[14] M. E. Jasin , “Nonsurgical Rhinoplasty Using Dermal Fillers,” Facial Plastic Surgery Clinics of North America 21, no. 2 (2013): 241–252. 23731585 10.1016/j.fsc.2013.02.004

[15] Y. Z. Chiang , G. Pierone , and F. Al‐Niaimi , “Dermal Fillers: Pathophysiology, Prevention and Treatment of Complications,” Journal of the European Academy of Dermatology and Venereology 31, no. 3 (2017): 405–413. 27662522 10.1111/jdv.13977

[16] E. Rahman , W. G. Philipp‐Dormston , W. R. Webb , et al., “‘Filler‐Associated Acute Stroke Syndrome’: Classification, Predictive Modelling of Hyaluronidase Efficacy, and Updated Case Review on Neurological and Visual Complications,” Aesthetic Plastic Surgery 48, no. 17 (2024): 3222–3253. 38971925 10.1007/s00266-024-04202-y

[17] D. Funt and T. Pavicic , “Dermal Fillers in Aesthetics: An Overview of Adverse Events and Treatment Approaches,” Clinical, Cosmetic and Investigational Dermatology 6 (2013): 295–316. 24363560 10.2147/CCID.S50546 PMC3865975

[18] F. Urdiales‐Gálvez , N. E. Delgado , V. Figueiredo , et al., “Preventing the Complications Associated With the Use of Dermal Fillers in Facial Aesthetic Procedures: An Expert Group Consensus Report,” Aesthetic Plastic Surgery 41, no. 3 (2017): 667–677. 28411354 10.1007/s00266-017-0798-y PMC5440530

[19] E. Currie , B. Granata , G. Goodman , et al., “The Use of Hyaluronidase in Aesthetic Practice: A Comparative Study of Practitioner Usage in Elective and Emergency Situations,” Aesthetic Surgery Journal 44, no. 6 (2024): 647–657. 38262634 10.1093/asj/sjae009 PMC11093658

[20] A. Borzabadi‐Farahani , A. Mosahebi , and D. Zargaran , “A Scoping Review of Hyaluronidase Use in Managing the Complications of Aesthetic Interventions,” Aesthetic Plastic Surgery 48, no. 6 (2024): 1193–1209. 36536092 10.1007/s00266-022-03207-9 PMC10999391

[21] G. Murray , C. Convery , L. Walker , and E. Davies , “Guideline for the Management of Hyaluronic Acid Filler‐Induced Vascular Occlusion,” Journal of Clinical and Aesthetic Dermatology 14, no. 5 (2021): E61–e69. PMC8211329 34188752

[22] M. Al‐Qattan and W. Al‐Kattan , “Skin Wound Healing, Ischemia‐Reperfusion Injury and Nerve Regeneration: Similarities in the Sequential Events and Molecular Basis,” Canadian Journal of Plastic Surgery = Journal Canadien de Chirurgie Plastique 12, no. 3 (2004): 131–133. 24115884 10.1177/229255030401200307 PMC3792800

[23] C. DeLorenzi , “New High Dose Pulsed Hyaluronidase Protocol for Hyaluronic Acid Filler Vascular Adverse Events,” Aesthetic Surgery Journal 37, no. 7 (2017): 814–825. 28333326 10.1093/asj/sjw251

[24] K. Beleznay , J. D. A. Carruthers , S. Humphrey , A. Carruthers , and D. Jones , “Update on Avoiding and Treating Blindness From Fillers: A Recent Review of the World Literature,” Aesthetic Surgery Journal 39, no. 6 (2019): 662–674. 30805636 10.1093/asj/sjz053

[25] H. Xiao , W. Kou , Y. Yang , et al., “Administration Method and Potential Efficacy of Hyaluronidase for Hyaluronic Acid Filler‐Related Vision Loss: A Systematic Review,” Aesthetic Plastic Surgery 48, no. 4 (2024): 709–718. 36574028 10.1007/s00266-022-03215-9

[26] L. O. Flores‐Salazar , S. E. Vázquez‐Lara , B. J. Moya‐Leal , et al., “Effect of Topical Nitroglycerin on the Survival of Random Pattern Vascular Skin Flaps in a Rat Model: Pilot Study,” Open Access Library Journal 10, no. 8 (2023): 1–10.

